# An integrated cell and medium engineering approach for production of a nanobody fusion in *Saccharomyces cerevisiae*

**DOI:** 10.1007/s00253-025-13700-1

**Published:** 2026-01-29

**Authors:** Laura R. K. Niemelä, Lotta-Mari Kirjavainen, Hendrikje C. J. Kozlowski, Heidi Salminen, Alexander D. Frey

**Affiliations:** Department of Bioproducts and Biosystems, Aalto University, Espoo, Finland

**Keywords:** *Saccharomyces cerevisiae*, Chemical chaperones, Small-scale production system, Medium optimization, Recombinant antibody production, Chimeric nanobody-Fc fusion protein

## Abstract

**Abstract:**

*Saccharomyces cerevisiae* is an established production host for therapeutic proteins; many of those are small proteins such as insulin or glucagon-like peptide-1 (GLP-1) analogs. Contrastingly, proteins of higher molecular weight, foremost antibodies, did not reach the market due, among other factors, to limiting productivity. Here we addressed the loss of product to protein degradation through a combination of genetic engineering of the host and medium optimization. We screened target genes that either directly or indirectly can lead to proteolytic degradation. We identified four deletions that are beneficial for expression: *PEP1* and *VPS30*, which both can channel proteins to the vacuole for degradation; *MON2*, which can lead to the re-uptake of secreted proteins; and *ALG3*, which can affect the permeability of the cell wall. In parallel, we developed a small-scale fed-batch cultivation system for 24-well deep well plate cultivations and using an amino acid-rich medium. To stabilize secreted proteins, we screened chemical chaperones and osmolytes. We fortified the medium with arginine, 4-phenylbutyrate (4-PBA), and Tween-20. Using the engineered yeast strain, which features *VPS30*, *PEP1*, and *ALG3* deletions, and the small-scale fed-batch system, we obtained 2.5 µg/mL of a secreted chimeric fusion of a nanobody to the crystallizable fragment (Fc) of a human immunoglobulin. Instrumental to the increase in the final titer were the reduced losses. This was achieved by a combination of complementary measures: improving diffusion through the cell wall, achieved through genetic engineering, and reducing losses to proteolytic degradation through medium optimization and genetic engineering. Moreover, we showed that the engineered strain and cultivation set-up are suitable for the production of different antibodies.

**Key points:**

• *Chemical chaperones and amino acid-rich medium increased secreted protein titers.*

• *Medium and host engineering are instrumental for improving productivity.*

• *Small-scale cultivation system enables production levels suitable for characterization.*

**Supplementary Information:**

The online version contains supplementary material available at 10.1007/s00253-025-13700-1.

## Introduction

*Saccharomyces cerevisiae* is an established production host for recombinant proteins, including proteins for therapeutic applications. In the year 2022, 38 biopharmaceuticals produced in *S*. *cerevisiae* were approved for the market in the United States and European Union (Walsh and Walsh 2022). Among those products were insulin and several insulin analogues, the B-isoform of the platelet-derived growth factor, several hepatitis vaccines including recombinant hepatitis B surface antigen, hirudin, human growth hormone, human GLP-1 receptor agonist, GLP-1 analog, and glucagon. A common denominator of most of these proteins is their rather small molecular weight, ranging between 3.4 kDa for GLP-1 analogs and 27 kDa for recombinant hepatitis B surface antigens, although exceptions to this commonality exist. These are human papilloma virus (HPV) vaccines composed of the major capsid protein (L1) of different HPV strains and the recombinant A-subunit of human factor XIII that have apparent molecular weights of 55 and 72 kDa, respectively, and indicate that larger proteins can also be produced with yields sufficient for commercialization. Despite a long history of research on the production of IgG-like molecules in different yeasts and fungal organisms and monoclonal antibodies and Fc fusion proteins representing the most rapidly growing class of biopharmaceuticals that dominate the biologics market, thus far, no antibodies produced in *S*. *cerevisiae* have entered the market (Joosten et al. 2003; Gasser and Mattanovich 2007; Walsh and Walsh 2022). Barriers for such products from *S*. *cerevisiae* include quality attributes such as the *N-*glycosylation pattern and productivity to be competitive with established production hosts.

Biological processes limiting productivity of biotherapeutics were identified along the whole secretory pathway, including translocation, protein folding, premature degradation, and intracellular miss-sorting of the target protein, but also metabolic limitations were considered as putative bottlenecks (Wang et al. 2017; Yang et al. 2024). Specifically, expression of monoclonal antibodies in *S*. *cerevisiae* benefitted from evolved signal sequences required for translocation, expression of molecular chaperones, expanding the volume of the endoplasmic reticulum (ER), and reducing losses to the ER-associated protein degradation system (Rakestraw et al. 2009; de Ruijter and Frey 2015; de Ruijter et al. 2016b; Niemelä et al. 2024). Some of those modifications were also beneficial for expression of single-chain variable fragment (scFv) or antigen-binding fragment (Fab fragment) in *S*. *cerevisiae* (Shusta et al. 1998; Xu et al. 2005; Xu and Robinson 2009). Thus, one of the major leverage points for boosting antibody productivity is the optimization of ER-localized processes of the secretory pathway, although the details of the required host engineering may differ depending on the expressed antibody format. Complementary research on recombinant protein production indicates that a significant fraction of the translated and folded polypeptides or of the secreted proteins can be degraded. While intracellular miss-sorting of proteins and degradation in the vacuole are well understood, also re-uptake of secreted proteins followed by degradation has been observed (Tyo et al. 2014; Rodríguez-Limas et al. 2015).

Heavy chain only antibodies originate from the *Camelidae* family (Hamers-Casterman et al. 1993), and in contrast to classical heterotetrametric immunoglobulins found in many other mammals, they lack the light chain (Muyldermans 2013; Arbabi-Ghahroudi 2017; Jin et al. 2023). Thus, in heavy chain antibodies, the antigen binding domain is formed by a single variable domain (VHH). Therefore, proper folding of the VHH domain does not require interaction with a second polypeptide, as is the case for assembling the antigen binding domain from two different variable domains, the V_H_ and V_L_ domains, respectively, in classical antibodies. As no intermolecular protein interactions are required for folding of the VHH domain, its surface is devoid of substantial hydrophobic patches. This feature reduces aggregation risk during folding and increases the solubility of the VHH domain (Bannas et al. 2017).

The discovery of the heavy chain antibodies enabled the construction of a new type of antibody fragment, nanobodies that comprise the VHH domain only (Desmyter et al. 1996). Nanobodies are well suitable as therapeutic and diagnostic agents (Jin et al. 2023). These small-sized molecules (< 15 kDa) are well expressed, have a high stability and solubility, and their secretion efficiency is higher compared to a single-chain variable fragment (scFv) molecule. Amongst other microbes, nanobodies were expressed in *S. cerevisiae* (Frenken et al. 2000; Thomassen et al. 2002; Wang et al. 2021). Nanobodies are well suitable for different modifications, including humanization to reduce immunogenicity by changing certain amino acids to more closely resemble the variable heavy chain domain of human antibodies (Bannas et al. 2017). Moreover, nanobodies can function as part of a fusion protein, for example, in cooperation with the crystallizable fragment (Fc) of a human immunoglobin, the Fc domain (Bell et al. 2010; Ji et al. 2013; Rotman et al. 2015; Qasemi et al. 2016). On the one hand, the additional Fc domain will impart effector functions to single-domain antibodies; on the other hand, it increases the serum half-life of single-domain antibodies due to the higher molecular weight evading glomerular filtration (Presta 2008).

In this work, we utilized an integrated approach to produce a chimeric nanobody–Fc fusion protein. To reduce losses to protein degradation, we utilized a two-pronged approach. We screened and identified genes that, when deleted, led to increased secreted antibody titers. The selected genes reduced intracellular miss-sorting, reuptake of secreted proteins by endocytosis, and increased permeability of the cell wall. We developed and used a small-scale fed-batch process for production. Amino acid-rich medium and a stable pH proved essential for prolonging the production phase and increasing secreted antibody titers. Moreover, we screened known small molecule effectors of protein folding and stability, among them commonly used osmolytes and chemical chaperones. Supplementation of the medium with selected compounds increased secreted antibody titers further. By utilizing the engineered strains and the established small-scale production scheme, we increased the final cell density by a factor of three and antibody titers by more than 20-fold compared to standard cultivation methods. The developed small-scale fed-batch protocol enables the production of antibodies at µg/mL scale sufficient for initial characterization.

## Materials and methods

Unless specified otherwise, all chemicals, reagents, and antibodies were purchased from Sigma-Aldrich (St. Louis, MO, USA), enzymes for molecular biology techniques from Thermo Fisher Scientific (Waltham, MA, USA).

### Antibody expression constructs

For expression of antibodies, four different plasmids based on plasmid pRS416-Gal1 were utilized (Mumberg et al. 1994). All constructs featured a *URA3* selection marker, the galactose inducible *GAL1* promoter (pGal1), and the antibody coding sequence. Different antibody formats were used in the expression tests: a camelid nanobody and a scFv, respectively, both featuring a human Fc domain appended to their C terminus, and two different tetrameric full-length antibodies. All antibody constructs contain the identical Fc region from human IgG_1_. Fusion of the Fc domain to the scFv and nanobody was carried out as described (Powers et al. 2001). All antibodies are described in Table 1. Details of the constructions of plasmids pLK1, pAX512, and pEK5 were described before (de Ruijter and Frey 2015; de Ruijter et al. 2017; Niemelä et al. 2024). Plasmid pCH1 is a derivative of pAX512 with the original Mat alpha signal sequence replaced with the signal sequence of the yeast *OST1* gene. Plasmid pCH1 was generated by amplifying the antibody from pCH1 with primers OAF55 and OAF57 (Supplemental Table S1), adding the signal sequence of the yeast *OST1* to the 5’ end of the antibody coding sequence. The resulting PCR product was inserted into *Spe*I and *Xho*I sites of plasmid pRS416-Gal1.

**Table 1:** Antibodies expressed in this study

Plasmid	Signal sequence	Antigen-binding domain	Target	Fcdomain
pLK1	Yeast OST1	VHH	Receptor-binding domain of SARS-CoV-2	Hinge-CH_2_-CH_3_ from human IgG_1_
pCH1	Yeast OST1	V_L_-V_H_	Hen egg lysozyme	Hinge-CH_2_-CH_3_ from human IgG_1_
pAX512	Yeast MAT alpha	V_L_-V_H_	Hen egg lysozyme	Hinge-CH_2_-CH_3_ from human IgG_1_
pEK5	Yeast PHO5	V_L_-C_L_ and V_H_-CH	CD20	Hinge-CH_2_-CH_3_ from human IgG_1_

### Generation of deletion strains

All strains used in this study were derived from *S. cerevisiae* W303α and are listed in Table 2. Target gene loci were deleted in W303alpha cells by replacement with PCR amplified deletion cassettes containing a dominant selection marker that was flanked by 50 base pairs homologous to the 5’ and 3’ ends of the target genes (Güldener et al. 1996).

**Table 2:** Yeast strains used in this study

Name	Genotype	Reference
W303α	*MAT*α {*leu2-3,112 trp1-1 can1-100 ura3-1 ade2-1 his3-11,15*}	ATCC 208353™
YEK18	*MAT*α {*leu2-3,112 trp1-1 can1-100 ura3-1 ade2-1 Δhis3:*(anti-CD20 antibody under GAL1:NatMX)	de Ruijter et al. 2016b
YEK13	*Δyps1::KanMX4*	This study
YEK12	*∆prb1::KanMX4*	This study
YLK1	*Δpep1::KanMX4*	This study
YLK2	*Δmon2::KanMX4*	This study
YLK3	*Δalg3::KanMX4*	This study
YLK4	*Δvps30::KanMX4*	This study
YLK5	*Δcym1::KanMX4*	This study
YLK6	*Δpep4::KanMX4*	This study
YHK4	*∆vps30*	This study
YHK5	*∆vps30∆pep1*	This study
YHK6	*∆vps30∆pep1∆mon2*	This study
YHK7	*∆vps30∆pep1∆alg3*	This study

Single deletion strains were generated using the KanMX4 cassette as a selection marker. Strains with multiple deletions were generated using the delete and repeat approach using the NatMX cassette (Hegemann and Heick 2011). Oligonucleotides used for the generation of strains with multiple deletions contained in addition *loxP* sites for the removal of the selection marker. All oligonucleotides were purchased from (Eurofins Genomics, Ebersberg, Germany) and are listed in Supplemental Table S1.

Deletion cassettes were generated using plasmids pUG6 or pUG74 (Güldener et al. 1996; Hegemann and Heick 2011), respectively, as templates and primer pairs listed in Supplemental Table S1. PCR reactions were carried out with Phusion High Fidelity DNA Polymerase according to manufacturers’ instructions.

PCR-amplified gene deletion modules were transformed into W303α by the lithium acetate method (Gietz and Schiestl 2007) and positive transformants were selected using YPD agar, supplemented with 200 μg/mL G418, or 100 μg/mL nourseothricin (Jena Bioscience, Jena, Germany).

To remove the gene disruption cassette from the genome, Cre-recombinase was expressed from plasmid pSH47 (Güldener et al. 1996) following established protocols (Hegemann and Heick 2011).

Removal of gene loci and removal of deletion marker were confirmed with colony PCR using KAPA2G PCR mix (KAPA Biosystems, Wilmington, MA, USA). Colonies were picked from a fresh agar plate and resuspended in 50 μL sterile ddH_2_O. The cell suspension was heated at 100 °C for 10 min, cooled down on ice, and centrifuged at 13,000*g* for 1 min. Confirmatory PCR was done with 4 μL of supernatant as template and oligonucleotide pair, with one specific for the target gene and one specific for the selection marker (Supplemental Table S1).

Expression plasmids were transferred into W303α by the lithium acetate method and transformants were selected on solidified synthetic drop-out medium lacking uracil (SD-URA) containing 2% glucose as carbon source. Colonies appeared on selective plates after 48 h of incubation at 30 °C.

### Small-scale batch cultivations

Small-scale batch cultivations were conducted in 24-well deep well plates with rectangular well design (Corning, NY, USA) on an orbital shaker at 30 °C and 220 rpm. Plates were covered with gas permeable sealing film during cultivations (BRAND® sealing film for microplates; Brand GmbH, Wertheim, Germany). Batch cultivations were performed in synthetic drop-out medium lacking uracil (SD-URA; Formedium Ltd., Norfolk, England) and containing 2% raffinose as carbon source. A single colony was picked from the transformation plates and used to inoculate 3 mL/well of selective medium. Plates were incubated for 24 h. Expression cultures were prepared by inoculation of 3.84 mL/well of selective expression medium fortified with 50 mM sodium phosphate buffer, pH 6.5, and 100 μg/mL bovine serum albumin (BSA) with pre-cultures to a starting OD_600_ of 0.2. Six hours after inoculation, expression of antibodies was induced by the addition of galactose to a final concentration of 0.5%, and plates were incubated for 16 h. Samples for measurements of optical cell density (OD_600_) and enzyme-linked immunosorbent assay (ELISA) were collected at the end of the cultivation.

### Testing of chemical chaperones as medium additives

The effects of medium additives were tested in small-scale batch cultivations as described above. The screen was performed using the antibody-expressing yeast strain YEK018 (de Ruijter et al. 2016b).

We selected 23 compounds described in the literature that act as chemical chaperones, help in the refolding of denatured proteins, or stabilize proteins and tested those for their effects in enhancing secreted antibody titers. The compounds included amino acids (alanine, arginine, glutamic acid, glycine, proline), detergents (Tween-20, Triton X-100), nondetergent sulfobetaines (dimethylethylammoniumpropane sulfonate (NDSB 195), (1-(3-sulfopropyl)pyridinium betaine (NDSB 201)), oxidants and reductants (dithiothreitol (DTT), diamide (tetramethylazodicarboxamide (TMAD))), divalent cations (CaCl_2_, MnCl_2_, CuSO_4_), glycerol, dimethylsulfoxide (DMSO), and sodium 4-phenylbutyrate (4-PBA), betaine, and trimethylamine *N*-oxide, taurine, adenosine triphosphate (ATP), and glucose-6-phosphate.

The compounds, and the concentration ranges used in the tests are listed in Supplemental Table S2. Four concentrations were tested per compound and their effects on final antibody titer and growth were determined as described above. Compounds exhibiting positive effects on titer, while not reducing growth, were more carefully tested, alone and in combination with others.

### Small-scale fed-batch cultivations

Small-scale fed-batch cultivations were conducted in 24-well deep well plates on an orbital shaker at 30 °C and 220 rpm. A single colony was picked from the transformation plates and used to inoculate 1 mL/well of selective preculture medium. Plates were covered with a breathable film and incubated for 48 h. After 48 h, secretion of soluble antibodies was initiated by the addition of a non-selective induction medium at a ratio of 2 mL/well of induction medium to 1 mL/well of preculture medium. Feed medium was added 24 and 48 h after induction at a rate of 0.7 mL/well/day. The pH of the cultures was adjusted with the addition of 1 M Na_2_CO_3_ at a rate of 50 µL/well/day.

Fifty-microliter samples were collected at the time of induction, addition of feed medium, and at the end of the cultivation for OD_600_ measurements. Samples were diluted in ddH_2_O in two steps: either 1:50 (precultures) or 1:100 (expression cultures) prior to OD_600_ measurements. Samples for ELISA and immunoblotting were collected 24 h and 48 h after induction.

Preculture medium for fed-batch cultivations was composed of 6.7 g/L yeast nitrogen base (YNB; Formedium Ltd., Norfolk, England), 4 g/L of amino acid dropout mix lacking uracil (CSM-URA; Formedium Ltd., Norfolk, England), 20 g/L glucose, and 10 mM sodium phosphate buffer, pH 6.3. Preculture medium was prepared from sterile stock solutions, a twofold concentrated YNB solution containing the dropout mix, a 200 mM phosphate buffer, pH 6.3, and a 40% glucose solution. Final concentration was adjusted with ddH_2_O.

The non-selective induction medium consisted of 8.33 g/L yeast nitrogen base, 16 g/L yeast extract, 32 g/L peptone, 6.8 g/L potassium phosphate monobasic, and 8.7 g/L potassium phosphate dibasic (50 mM phosphate buffer), and 50 g/L galactose, with the pH adjusted to 6.3 with 3 M HCl. The medium was sterilized through a 0.22 µm filter. The medium was supplemented with 0.0025% Tween-20 and 15 mM arginine.

### Determination of antibody titers

Samples were prepared by clearing cultures by centrifugation for 3 min at 20,900*g* and 4 °C. The cleared culture supernatants were diluted 1:6 with 1 × PBT (135 mM NaCl, 2.5 mM KCl, 10 mMNa_2_HPO_4_, 1.75 mM KH_2_PO_4_) + 0.05% Tween-20) and were stored at − 20 °C. Titers of the secreted antibodies were determined using an ELISA with antibodies for binding and detection specific for the Fc domain as described earlier (de Ruijter and Frey 2015). For verifying the presence of the human V_L_-V_H_ sequences, the same procedure was followed except that a 1:4000 dilution of goat anti-human kappa light chains (bound and free) peroxidase-labeled antibody was used for detection. Due to the lack of a suitable standard antibody, the V_L_-V_H_ titers are expressed in units of A_490_.

### Lysozyme binding assay

An ELISA was used to measure antigen-binding activity. ELISA plates were coated with 100 μL/well of 5 μg/mL lysozyme from chicken egg white in 0.05 M sodium carbonate buffer (1.515 g/L Na_2_CO_3_, 3 g/L NaHCO_3_, pH 9.6) overnight at 4 °C. After removal of the coating solution, plates were washed four times with 200 μL PBT and blocked for 45 min in PBT at 22 °C with occasional shaking. 200 μL of samples in triplicates were added to the wells, and the plates were incubated for 90 min while shaking at room temperature. After incubation, wells were washed five times with 200 μL of PBT. 100 μL/well of 1:4000 dilution of goat anti-human IgG (Fc specific) peroxidase-labeled antibody was added and allowed to bind for 1 h at room temperature while shaking. After incubation, wells were washed five times with 200 μL of PBT. For detection, 100 μL/well substrate solution (0.2 mg/mL o-phenylenediamine, 3 μL of 30% H_2_O_2_ per 10 mL solution in 0.05 M phosphate citrate buffer, pH 5) was added. After 8 min, the reaction was quenched by the addition of 100 μL/well of 3 M H_2_SO_4_. Absorbance was read at 490 nm using a BioTek Synergy 2 spectrophotometer. Data evaluation was done with Gen5 software (Biotek Instruments, Winooski, VT, USA). Due to the lack of a suitable standard antibody, the lysozyme binding is expressed in units of A_490_.

### Immunoblot analysis

The stability and integrity of secreted antibodies in the culture supernatants was verified using immunoblotting. Cleared culture supernatants were prepared as described above, and reduced and non-reduced protein samples were produced thereof. Non-reduced protein samples were obtained by mixing 75 µL of culture supernatant with 25 µL of 4 × SDS sample buffer. Reduced protein samples were obtained likewise by adding 5 µL of 1 M DTT prior to heating. Samples were incubated for 10 min at 65 °C and cooled on ice. Twenty microliters of reduced protein samples were deglycosylated with 0.5 µL of endoglycosidase H (EndoH) (New England Biolabs, Ipswich, MA, USA) as described to verify the *N-*glycosylation status (Piirainen and Frey 2020). Twenty microliters of either non-reduced, reduced, or reduced and EndoH-treated protein samples were resolved on either 7.5% SDS-PAGE or 10% SDS-PAGE gels, respectively. As a positive control, 5 µL of a 10 ng/µL reduced standard human IgG_1_ antibody were loaded.

SDS-PAGE gels were run according to standard protocols and the resolved proteins were transferred to nitrocellulose membranes. Incubation of membranes and detection of antibodies was carried out as described using an anti-human IgG HRP conjugate diluted 1:4000 for detection (Piirainen and Frey 2020).

### Data processing

All numeric data was analyzed using GraphPad Prism 6 (GraphPad, San Diego, CA, USA). Ordinary one-way ANOVA followed by Dunnett’s multiple comparison test was applied.

## Results

### Screening target genes for reducing losses to proteolytic degradation

We selected several target genes that were implicated or were shown to improve heterologous protein expression in yeast by reducing losses to proteolytic degradation, either by direct or indirect action. Four of the selected target genes were proteases encoded by *CYM1*, *PEP4*, *YPS1*, and *PRB1*. The remaining genes indirectly lead to proteolytic degradation, either by intracellular mis-sorting to the vacuole (*PEP1*, *VPS30*) or re-uptake of secreted proteins by endocytosis (*MON2*). Finally, we selected *ALG3*, which is involved in lipid-linked oligosaccharide synthesis; however, its deletion also affects the permeability of the cell wall and could support diffusion through the cell wall. We deleted the target genes in the wild-type strain W303α, and the resulting deletion strains were transformed with plasmid pLK1.

We grew the strains in standard yeast cultivation medium that contained the non-repressing carbon source raffinose. This set-up allowed the induction of the promoter by galactose addition at the optimal time point. Furthermore, we supplemented the medium with BSA to stabilize the secreted chimeric nanobody–Fc fusion during the cultivation (Rakestraw et al. 2009). Final cell density and secreted antibody titers were determined 16 h after induction.

**Fig. 1:**
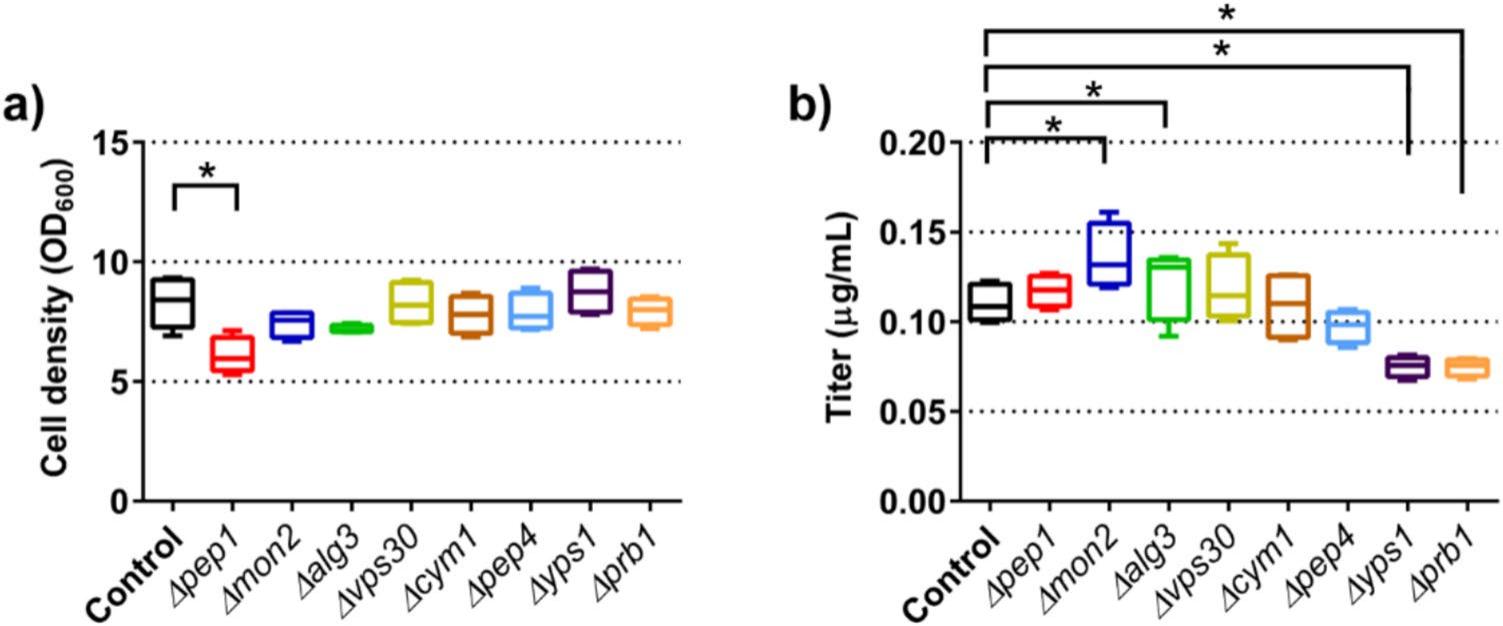
Effects of deleting the selected target genes on final cell densities (**a**) and secreted antibody titers (**b**). Yeast deletion strains and their parental strain (control) expressing chimeric nanobody-Fc fusion were grown in 24-well deep well plates using the small-scale batch protocol. Final cell densities and titers of secreted chimeric nanobody-Fc fusion were determined 16 h after induction. The data is depicted as the full range, quartiles, and median of five to seven biological replicates. *P*-values were calculated by pairwise comparison of deletion strains to the control strain. *: *P* < 0.05; **: *P* < 0.01; ***: *P* < 0.001

Final cell densities of deletion strains varied between 88 and 107% compared to the control strain, except for the *PEP1* deletion strain (Fig. 1a). This deletion strain reached only 74% of the cell density compared to the control. Despite numerous reports that deletion of protease genes increases productivity, none of the four protease deletion strains accumulated higher amounts of secreted antibodies, and for *YPS1* and *PRB1* deletion strains, significant reductions were observed (Fig. 1b). In contrast, deletion of the four other genes resulted in small increases in the secreted antibody titers. Among those strains, the *MON2* deletion strain exhibited the highest increase, reaching 23% higher antibody levels, while the other strains showed small increases. However, as most of these strains had a small growth phenotype, OD_600_ standardized antibody titers increased by around 40% in the case of *PEP1* and *MON2* deletion strains and by 25% in the case of the *ALG3* deletion strain, respectively (data not shown).

### Evaluating the effect of chemical chaperones on growth and antibody production

Comparably few reports exist on how chemical chaperons or osmolytes can benefit recombinant protein production, although their utilization for increasing production has been postulated almost 30 years ago (Welch and Brown 1996). Chemical chaperones can improve limitations in intracellular protein trafficking and minimize protein aggregation, thus relieve ER stress (Roth et al. 2012). Osmolytes are small molecules that help stabilize protein structure and promote proper folding, especially under conditions that might otherwise lead to denaturation. Osmolytes can be distinguished further by their mode of action (Street et al. 2006; Abe et al. 2015). As antibody folding requires formation of multiple disulfide bonds, we included also the reductant DTT and the oxidant diamide that can potentially modulate the redox potential (Frand and Kaiser 1998). The effects of medium additives were tested in small-scale batch cultivations. Concentrations of the medium additives were estimated from literature reports that covered a wide range of experimental designs, including studies on recombinant protein expression as well as in vitro protein folding (Supplemental Table S2). In the first round of testing, we selected one concentration that was higher and one concentration that was lower than the reported concentration. In the second round, the compounds were evaluated at two additional concentration levels that were set above and below the concentration level that was performing better. We rated compounds based on the observed effects on growth and antibody production (Supplemental Table S2). None of the tested substances had a positive effect on final cell density, and most of the compounds led to reduced cell densities after 16 h of expression. The effects of the compounds on antibody titers were more variable; similar numbers of compounds led to either small to moderate increases or decreases in antibody titers. Furthermore, one fourth of the compounds did not produce any effects (Supplemental Table S2).

Out of the 23 compounds included in the screen, three compounds which did not negatively impact growth and increased antibody titers were selected for further optimizations. These compounds were arginine, the non-ionic surfactant Tween-20, and 4-PBA. Arginine was reported earlier to reduce post-secretion loss to proteolytic degradation (Kang et al. 2000). Tween-20 and other non-ionic detergents were shown to increase secreted protein levels (Baumann et al. 2011). 4-PBA is a chemical chaperone that has been shown to prevent the activation of the unfolded protein response (UPR) in in vitro and in vivo studies and to increase antibody titers in mammalian cell cultures (Ayala et al. 2012; Roth et al. 2012; Johari et al. 2015; Mai et al. 2018).

**Fig. 2:**
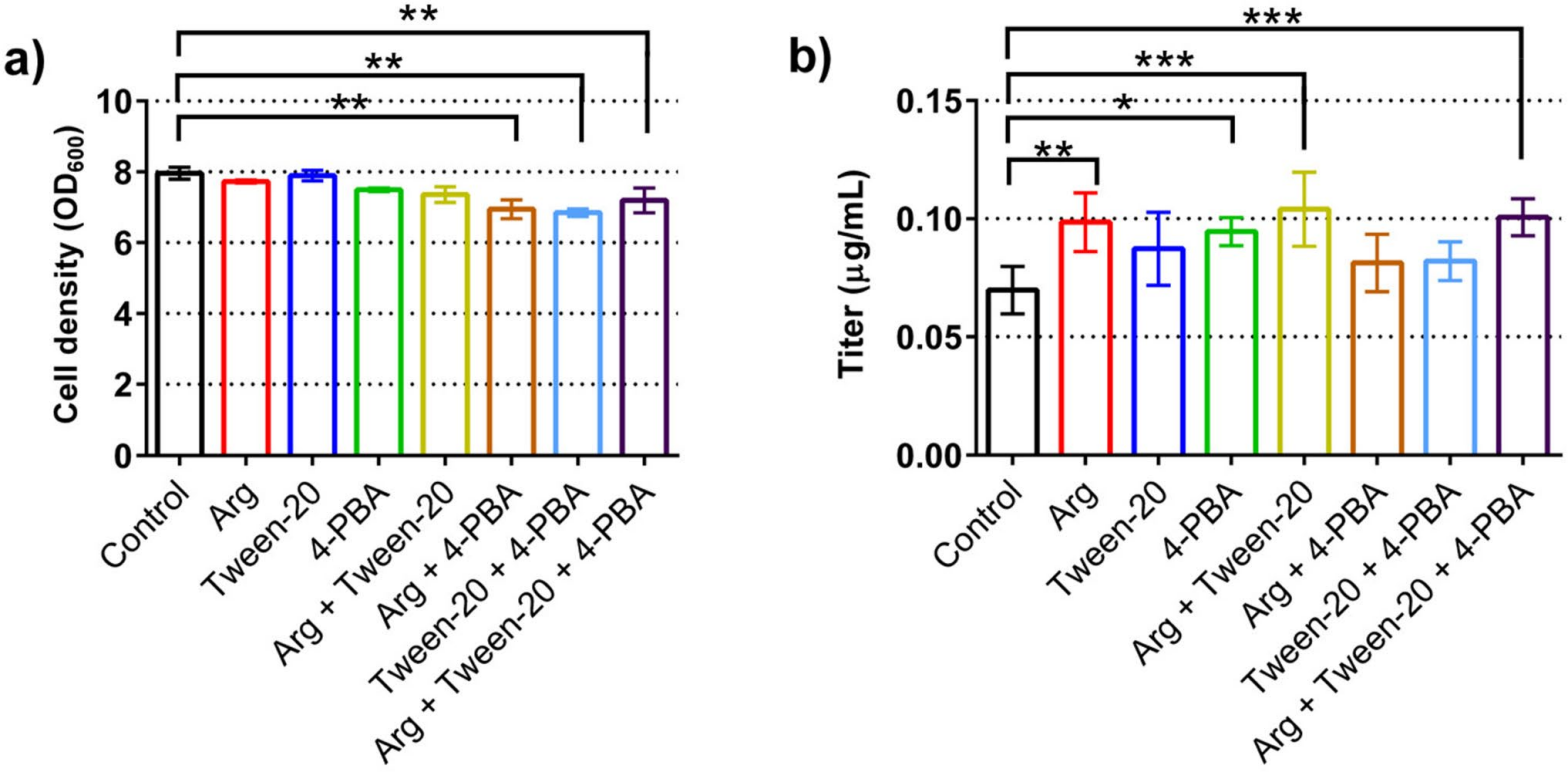
Effects of chemical chaperones on final cell density (**a**) and secreted antibody titers (**b**). The yeast strain YEK18 expressing a full-length antibody was used for the tests and was grown in 24-well deep well plates using the small-scale batch protocol. Cultures were supplemented with 15 mM arginine, 0.0025% Tween-20, and 0.25 mM 4-PBA, alone or in the indicated combinations or were left untreated (control). Final cell densities and titers of secreted full-length antibody were determined 16 h after induction of expression. The data are presented as mean and standard deviation of three biological replicates. *P*-values were calculated by pairwise comparison of deletion strains to the control strain. *: *P* < 0.05; **: *P* < 0.01; ***: *P* < 0.001

We evaluated those compounds, alone or in combinations, more carefully. When used alone, all compounds led to small decreases in final cell densities that became more accentuated when used in combinations (Fig. 2a). Out of three compounds, 4-PBA had the strongest negative impact on growth that was seen when used alone or in combination with the other compounds.

All three compounds significantly increased the final antibody titers, reaching up to 40% higher concentrations compared to the control (Fig. 2a). When testing the combinations, arginine and Tween-20 had the highest impact on antibody concentrations; however, the increase compared to the best performing compound used alone was small (Fig. 2b). Notably, while 4-PBA was effective when used alone, when used in combination with arginine or Tween-20, it seemed to reduce the effect of the other compound. Therefore, we selected arginine and Tween-20 at concentrations of 15 mM and 0.0025%, respectively, for further experiments.

### Establishing a small-scale fed-batch protocol for antibody production

Finally, we aimed to set up a small-scale fed-batch system that allows for the production of secreted antibody in quantities sufficient for the initial characterization of the produced molecules. For example, *N-*glycans can be isolated from proteins and analyzed by MALDI-TOF MS from quantities as small as 5 µg. For this purpose, we developed a cultivation system that included a batch phase in selective medium to produce biomass, followed by a production phase in a rich, non-selective medium that should prevent proteolytic degradation and minimize post-secretion loss. We estimated that during the production phase, growth would be limited, resulting in two to three doublings of the cell density; thus, the risk of plasmid loss would be negligible.

The concept and initial parameters for the fed-batch process and the medium composition were taken from an earlier publication (Wec et al. 2020), but we aimed to simplify the system. In a first step, we optimized the preculture and induction medium. We tested preculture medium with different amounts of glucose (0.5, 2, and 4%) and phosphate buffer (0, 10, and 50 mM), while keeping the composition of the initial induction medium constant. These experiments showed that 2% glucose and 10 mM phosphate buffer were optimal for the preculture medium to reach maximal productivity. Notably, we observed that using the higher phosphate concentration during preculture led to an increased amount of degraded antibody molecules, as judged from immunoblotting analysis (data not shown).

**Fig. 3:**
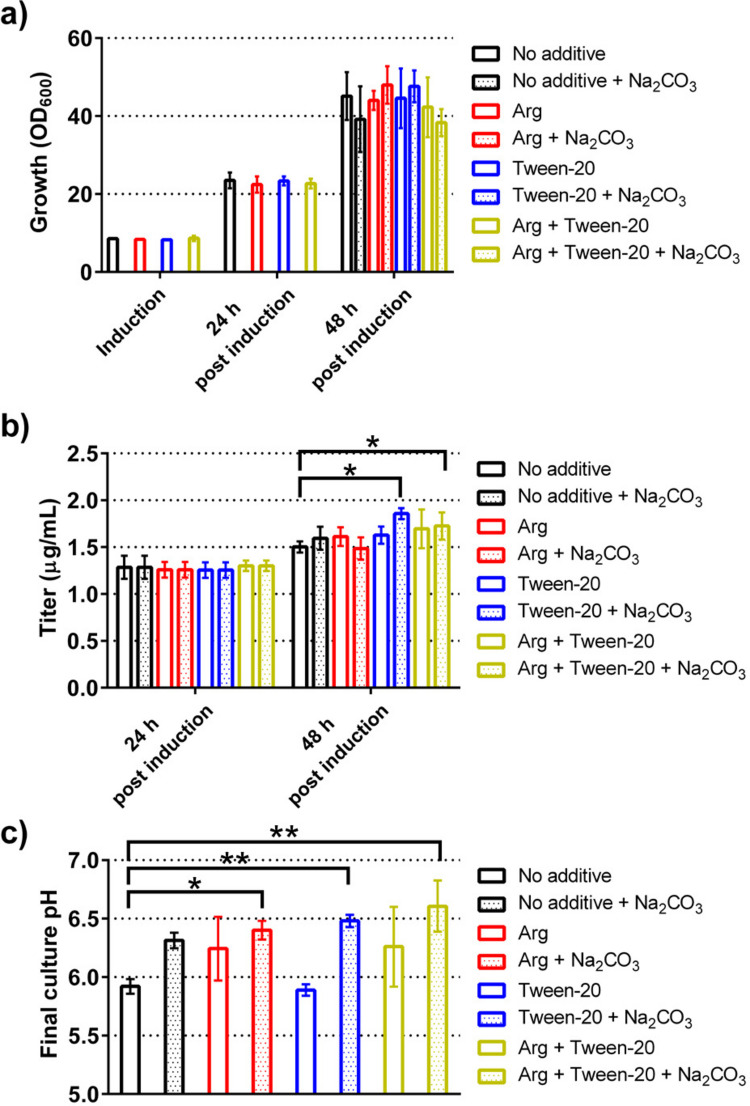
Establishing small-scale fed-batch cultivation protocol. A wild-type yeast strain expressing chimeric nanobody–Fc fusion was grown in 24-well deep well plates. Culture medium was supplemented with 15 mM arginine, 0.0025% Tween-20, or a combination of both. Cultures were induced with induction medium after 48 h of preculture. The culture pH was readjusted by the addition of 50 µL of 1 M Na_2_CO_3_ 24 h after induction. Cell densities were determined before induction and 24 and 48 h after induction (**a**). Titers of secreted chimeric nanobody–Fc fusion (**b**) were determined 24 and 48 h after induction. The pH of the culture medium was measured at the end of the cultivations (**c**). The data is presented as mean and standard deviation of three biological replicates. *P*-values were calculated by pairwise comparison of cultivation conditions to the control (No additive). *: *P* < 0.05; **: *P* < 0.01; ***: *P* < 0.001

The cell density after preculture was 8.48 ± 0.34 OD_600_ and reached 23.39 ± 1.15 OD_600_ and 45.12 ± 6.14 OD_600_, 24 h and 48 h post-induction (Fig. 3a). Thus, as assumed, the biomass formation during the production phase was modest. The observed titers reached 1.28 ± 0.12 µg/mL and 1.50 ± 0.06 µg/mL, 24 and 48 h post-induction, which is tenfold higher compared to the titers reached earlier (Fig. 1a). The addition of Tween-20, arginine, or the combination of both to the induction medium did result in increases in the final titers in the range of 20%, the effects manifesting only 48 h post-induction, and the titer reached up to 1.70 ± 0.21 µg/mL when using medium containing Tween-20 and arginine (Fig. 3b). Furthermore, we evaluated the addition of the induction medium at a rate of 0.7 mL/day or the addition of a feed medium consisting of YNB and galactose at a rate of 0.7 mL/day 24 and 48 h post-induction during the production phase. Neither of the treatments led to increased antibody titers after 48 or 72 h after induction (data not shown). As we observed a drop of pH below pH 6 during the production phase, we also evaluated the effect of pH adjustment on production. The addition of 50 µL/day of 1 M Na_2_CO_3_ proved sufficient to maintain the pH at the target value of 6.3 to 6.5. Furthermore, the addition of 15 mM arginine to the culture medium alone had a beneficial effect on culture pH (Fig. 3c); however, the change was not statistically significant. Besides the direct effect of stabilizing the pH, the addition of Na_2_CO_3_ also led to small increases in the final antibody titer when used together with either Tween-20 or arginine and Tween-20.

**Fig. 4:**
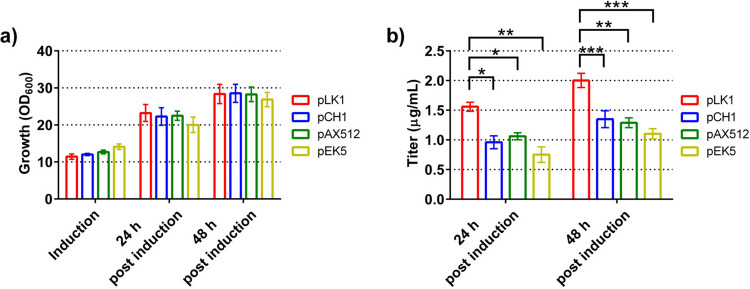
Production of different antibodies using the small-scale fed-batch cultivation system. The optimized cultivation system was used to cultivate wild-type yeast cells expressing antibodies representing different antibody formats: pLK1: chimeric nanobody-Fc fusion; pCH1: chimeric scFv-Fc fusion; pAX512: chimeric scFv-Fc fusion; pEK5: full-length IgG. Cell densities were determined before induction and 24 and 48 h after induction (**a**). Titers of secreted antibodies (**b**) were determined 24 and 48 h after induction. The data is presented as mean and standard deviation of three biological replicates. *P*-values were calculated by pairwise comparison of all strains. *: *P* < 0.05; **: *P* < 0.01; ***: *P* < 0.001

Finally, we verified that the developed protocol would be suitable also for the production of other antibody formats. For this purpose, we used wild-type yeast and expressed four different antibody constructs. We observed that the growth of all four expression strains was similar, reaching a final OD_600_ of close to 30 after 48 h of production (Fig. 4a). The secreted antibody titers reached up to 2.00 µg/mL after 48 h of expression (Fig. 4b). The titers of secreted antibody varied; the titers of the full-length monoclonal antibodies were lowest, reaching 1.10 µg/mL, while the chimeric scFv-Fc fusions reached titers of 1.29 and 1.35 µg/mL, respectively. The structurally least complex antibody format, the chimeric nanobody-Fc fusion, reached titers of 2.00 µg/mL. Overall, the developed expression system allowed for the production of highly increased antibody titers stemming from a compound effect, improved and prolonged growth, and increased specific productivity.

### An optimized production strain improves antibody titers

Next, based on the results from the initial screen, we generated a yeast strain where we deleted the genes that proved to be most effective in enhancing antibody productivity (Fig. 1a); thus, we selected *VPS30*, *MON2*, *PEP1*, and *ALG3*. Using the delete and repeat approach, marker-free expression strains were generated, and the resulting strains were transformed with the expression construct for the chimeric nanobody–Fc fusion.

**Fig. 5:**
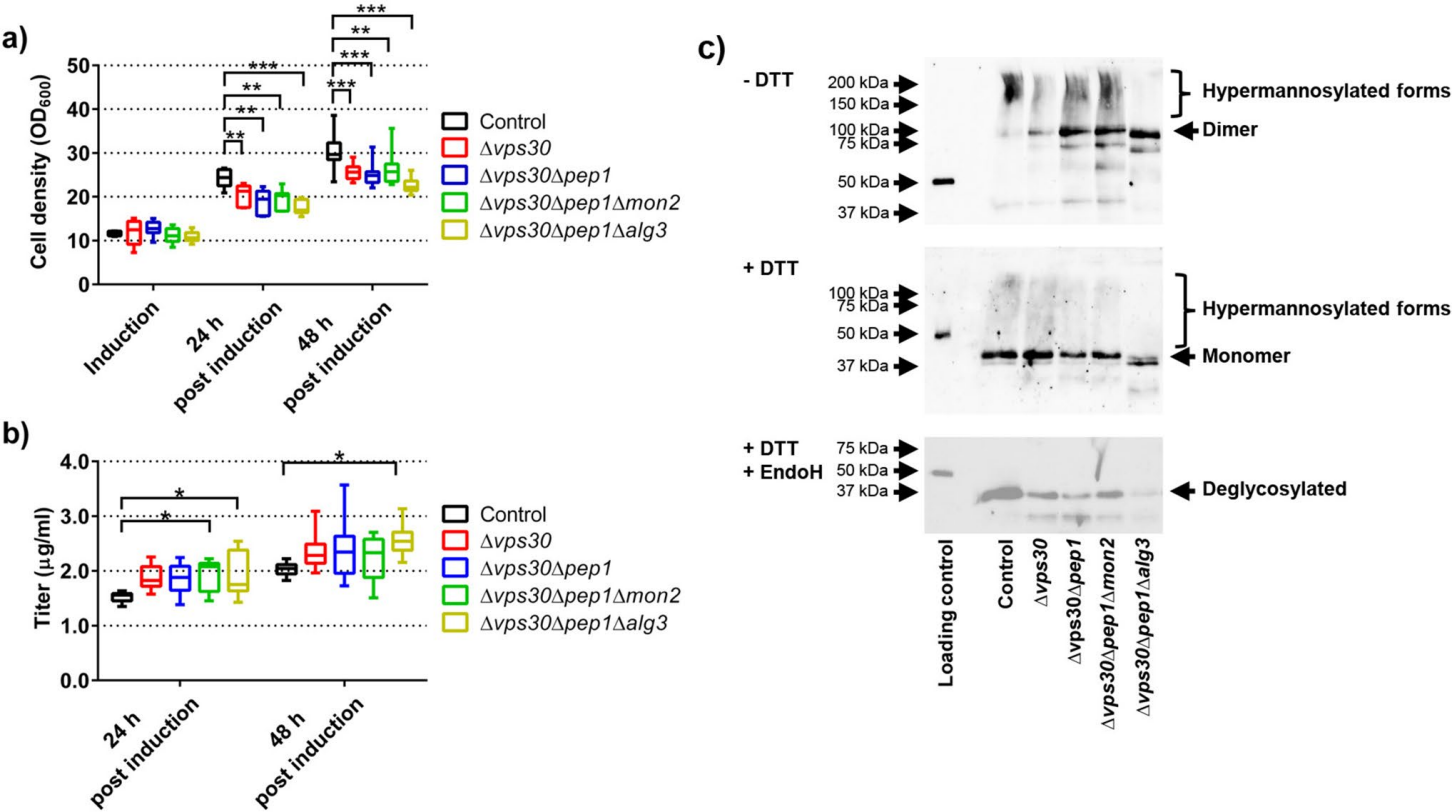
Cell engineering enhances chimeric nanobody-Fc fusion production with the small-scale fed-batch cultivation system. Four different deletion strains were transformed with the plasmid for expression of the chimeric nanobody-Fc fusion construct. Yeast deletion strains and their parental strain (control) expressing chimeric nanobody-Fc fusion were grown in 24-well deep well plates using the small-scale fed-batch cultivation system. Cell densities were determined before induction and 24 and 48 h after induction (**a**). Titers of secreted chimeric nanobody-Fc fusion (**b**) were determined 24 and 48 h after induction. (**c**) Immunoblot analysis of chimeric nanobody-Fc fusion proteins expressed in control and deletion yeast strains. Non-reduced (- DTT), reduced (+ DTT) and reduced deglycosylated (+ DTT + EndoH) samples were resolved on SDS-PAGE gels and detected using an antibody specific for the Fc domain of human IgG_1_. In the strain containing the *ALG3* deletion, a mobility shift towards a lower molecular weight compared to the other strains is visible; this shift represents the change in the N-glycosylation pattern caused by the *ALG3* deletion. Hyperglycosylated forms of the expressed antibody are indicated with brackets. The loading control is the heavy chain of a reduced human IgG_1_ antibody. The data is depicted as the full range, quartiles and median of six to twelve biological replicates. *P*-values were calculated by pairwise comparison of deletion strains to the control strain. *: *P* < 0.05; **: *P* < 0.01; ***: *P* < 0.001

Deletion of the genes resulted in a reduction of the final cell densities compared to the control, reaching on average 75 to 85% of the final cell density of the control. Moreover, these differences resulted mostly in the first 24 h after induction. As is obvious, the negative effects of the deletions only materialized during the production phase, as at the time of induction, no significant differences in cell density were apparent (Fig. 5a). Average antibody titers were up to 25% higher in the deletion strains compared to the control, reaching 2.55 µg/mL with the Δ*VPS30*Δ*PEP1*Δ*ALG3* deletion strain. Although there is no direct link between *ALG3* and productivity, the strain with the additional *ALG3* deletion performed slightly better than its parental strain Δ*VPS30*Δ*PEP1*, reaching 2.40 µg/mL. Overall, the antibody titers reached with the optimized cultivation set-up were 20-fold higher compared to the titers obtained with standard cultivation medium (Fig. 1), even though the standard cultivation medium was fortified with additional buffering capacity and BSA to stabilize secreted proteins. Furthermore, secreted antibody titers increased over the 48-h production phase (Fig. 5b). In contrast, when using standard cultivation medium, most secreted antibodies were degraded after longer incubation times (data not shown).

Finally, we verified that the expressed proteins were intact. For this, we sampled culture supernatants at the end of the cultivations. We prepared non-reduced and reduced samples and analyzed the proteins using immunoblotting after they were resolved by gel electrophoresis. In addition, to verify the *N*-glycosylation, we enzymatically deglycosylated the reduced samples. The results show that the chimeric nanobody–Fc fusions were correctly produced as a dimer that can be reduced, generating the monomeric form of the expected molecular weight (Fig. 5c, Supplemental Fig. S1). Furthermore, while the control strain and three of the deletion strains produced the protein in the hypermannosylated form typical for *S*. *cerevisiae*, deletion of the *ALG3* reduced the *N*-glycan size and heterogeneity and produced an *N*-glycan that is compatible with different glycoengineering approaches (Choi et al. 2003; Piirainen et al. 2021).

### Yeast cells produce functional antibodies that bind lysozyme

**Fig. 6:**
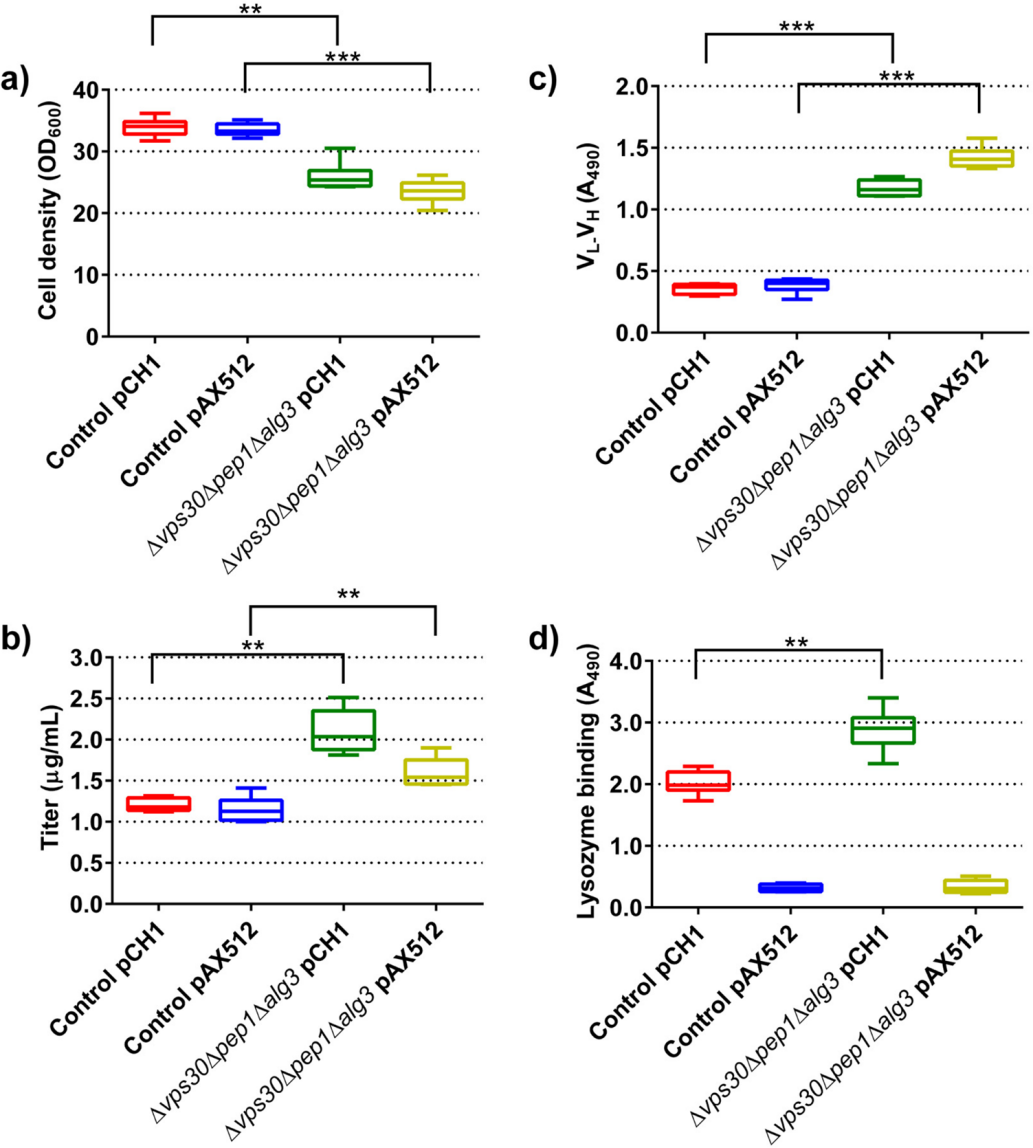
Production of lysozyme binding antibodies using the small-scale fed-batch cultivation system. The optimized cultivation system was used to cultivate wildtype and Δ*VPS30*Δ*PEP1*Δ*ALG3* yeast cells expressing two chimeric scFv-Fc fusion antibodies from plasmids pCH1 and pAX512. Cell densities were determined before induction and 48 h after induction (**a**). Titers of secreted chimeric scFv-Fc fusion (**b**) were determined 48 h after induction. An Fc-LC ELISA (**c**) and antigen-Fc ELISA (**d**) were used to determine the integrity and functionality of the produced antibodies. The data is depicted as the full range, quartiles, and median of six biological replicates. *P*-values were calculated by pairwise comparison of the strains. *: *P* < 0.05; **: *P* < 0.01; ***: *P* < 0.001

We selected the control strain and the Δ*VPS30*Δ*PEP1*Δ*ALG3* strain and transformed them with two plasmids (pCH1 and pAX512) producing an antibody directed against lysozyme from hen egg white to test whether a functional antibody is produced. Utilizing the optimized cultivation scheme, we collected supernatants from the strains 48 h post-induction. We observed the well-known growth phenotype; both control strains reached significantly higher final cell densities than the engineered strains (Fig. 6a). We subjected the samples to three different ELISA tests. First, the standard ELISA was used to determine titers where capture and detection of the antibody is mediated via the Fc domain (Fc-Fc ELISA). Secreted titers were in the range of the previous experiments, and they were significantly higher when the antibodies were expressed from the engineered Δ*VPS30*Δ*PEP1*Δ*ALG3* strains compared to the control strains (Fig. 6b). Next, we verified that the produced antibodies were intact and functional. We used an ELISA where the produced antibodies are captured via the Fc-domain and detected via the detection antibody specific for the kappa light chain (LC) of the engineered V_L_-V_H_ domain (Fc-LC ELISA), and an ELISA where the antibody is captured via its antigen lysozyme and detected via its Fc-domain (antigen-Fc ELISA). These two ELISAs were performed semi-quantitatively due to the lack of corresponding control antibodies. According to the Fc-LC ELISA, all four strains produced antibodies, and the engineered strains produced a significantly higher amount compared to the control strains, reflecting the results of the Fc-Fc ELISA. Next, we tested whether the produced antibodies would bind to lysozyme. After coating the plates with lysozyme overnight and blocking, supernatants were applied and plates incubated. After thorough washing, the captured antibodies were detected. The antigen-Fc ELISA revealed a strong signal for the antibody expressed from pCH1 that was significantly stronger when the antibody was expressed from the Δ*VPS30*Δ*PEP1*Δ*ALG3* strain compared to the control strain (Fig. 6d). Interestingly, the antibody produced from pAX512 showed very little signal when applied at the same dilution (1:6) as the antibody produced from pCH1, irrespective of the strain with which the antibody was produced. Further experiments revealed that less diluted supernatants were required to produce significant signals with the antigen-Fc ELISA compared to the antibody produced from pCH1 (data not shown).

## Discussion

All genetic modifications tested aimed at reducing losses due to proteolytic degradation, miss-sorting, and endocytosis. In contrast to previous reports, deletion of protease genes did not have any positive effect on antibody titers. In contrast, gene deletions targeting intracellular sorting (*VPS30* and *PEP1*), endocytosis (*MON2*), and *N-gl*ycosylation (*ALG3*) had a positive effect on secreted antibody titers.

Deletion of the *PEP1* gene was earlier shown to prevent miss-sorting of recombinant proteins featuring the mating factor alpha pre- and prosequences in both *S*. *cerevisiae* and *Pichia pastoris* into the vacuole (Fitzgerald and Glick 2014). However, a side effect of the deletion of *PEP1* is the secretion of vacuolar proteins to the culture medium, a consequence that can be mitigated by using only a partial deletion of *PEP1*. Furthermore, we had identified *VPS30* in a genetic screen for factors improving antibody production (de Ruijter et al. 2016a); thus, the additional results further corroborate that deletion of *VPS30* can improve recombinant protein production, in particular, the expression of antibodies.

In yeast cells, when a protein is exocytosed through the plasma membrane, the protein is entrapped in the periplasm, i.e., the space delimited by the plasma membrane and cell wall. Tyo et al. (2014) suggested that if a recombinant protein is secreted into the periplasm, the protein can either (a) diffuse through the cell wall to the medium or (b) be endocytosed. The authors underlined this statement by showing that *S*. *cerevisiae* can consume protein that was supplemented to the medium at a rate of 1 g/L/day (Tyo et al. 2014). Consequently, increasing the diffusion through the cell wall should decrease endocytosis. As the cell wall proteins are often highly *N*-glycosylated, it is conceivable that *ALG3* deletion leads to an altered cell wall structure that is less dense, resulting in a better diffusion of secreted antibody from the periplasm through the cell wall. This hypothesis is also supported by findings showing that deletion of the *ALG3* gene in combination with the *OCH1* gene strongly affects the cell wall structure and renders the mannoprotein layer thinner (Xu et al. 2016), which should increase the permeability of the cell wall for secreted proteins.

Another aspect regarding the *ALG3* deletion is noteworthy - —deletion of *ALG3* is a cornerstone in many glycoengineering strategies in *S*. *cerevisiae* and other yeasts or fungi (Choi et al. 2003; Piirainen et al. 2014, 2021). Thus, creating a yeast strain with *ALG3* deletion ensures compatibility with glycoengineering approaches. We also showed that the mobility on SDS-PAGE gels of the antibody produced with the Δ*VPS30*Δ*PEP1*Δ*ALG3* deletion strain changed compared to the antibodies produced in the other strains, indicating that the *ALG3* deletion also altered the *N*-glycosylation profile.

Our results also show that reducing endocytosis through deletion of the *MON2* gene increased antibody titers. Mon2p, also designated Ysl2p, has a function in endocytosis and maintenance of vacuole integrity for which it interacts with the Arf-like small GTPase Arl1p by acting as a negative regulator. *MON2* deletion cells were not impaired in the kinetics of alpha-factor internalization at the plasma membrane, but they exhibited a strong delay in alpha-factor turnover. Furthermore, a model compound for endocytosis, the dye lucifer yellow, was only weakly staining the vacuoles of the mutant cells (Jochum et al. 2002). Other genes, such as *END3* and *RVS161*, involved in endocytosis have been assessed for their contribution in the turn-over of secreted proteins, α-amylase, a strongly glycosylated protein of 54.8 kDA, and the *N*-glycosylated insulin precursor protein with a molecular weight of 12.0 kDA. The positive effects on protein accumulation were only seen for α-amylase, but not for the insulin precursor, leading the authors to conclude that the small insulin precursor protein more readily diffuses through the membrane than the larger amylase (Rodríguez-Limas et al. 2015). Here, we expressed proteins with nominal molecular weights ranging from 78.7 kDa to 150 kDa; thus, diffusion through the cell wall would be considerably impaired compared to small proteins such as the insulin precursor protein.

A key aspect for improving secreted antibody titers was the development of a medium that improved the specific productivity through minimizing losses to proteolytic degradation. This was achieved by utilizing an amino acid-rich medium and maintaining the culture medium above pH 6.3. The importance of a controlled pH environment on the stability of certain secreted proteins is well known, and several solutions to this problem can be applied, including buffering the culture medium, adjusting the pH with base, or complementing the medium with amino acid-rich components. In this work, we applied all three strategies to maintain pH above 6.3. We also noticed that the addition of arginine had a positive effect on the final culture medium. Kang et al. (2000) observed that the addition of 100 to 300 mM arginine reduced the degradation of human serum albumin (HSA) in yeast culture medium. The authors noted that arginine did not stabilize pH but suggested that the supplementation would repress the expression of proteases responsible for the degradation of HSA (Kang et al. 2000). In our work, the optimized concentration for arginine was considerably lower than the concentration shown to protect HAS in yeast cultures (Kang et al. 2000).

Furthermore, we systematically screened known molecular chaperones and osmolytes for their effect on antibody production. From the molecules tested, 4-PBA, arginine, and Tween-20 were the most successful compounds for increasing antibody titers, and the latter two compounds were included in the final medium as they also had additive effects, while 4-PBA was omitted as it did not have any synergistic effects. However, 0.25 mM 4-PBA, when used alone, would be a promising compound for increasing the concentration of secreted antibodies. Notably, 4-PBA is used in mammalian cell culture for improving productivity, where it is believed to act as a chemical chaperone that mitigates ER stress and induction of the UPR in CHO cells expressing antibodies and thus complements the function of molecular chaperones (Johari et al. 2015). However, research points out that the observed mitigation of ER stress in *S*. *cerevisiae* by 4-PBA is a result of increased degradation of the ER-stress sensor Ire1p (Mai et al. 2018). Therefore, the increased antibody titers observed in this study might be rather due to the attenuated UPR than the function of 4-PBA as a molecular chaperone. UPR induction is, for cells, an energetically demanding and resource-intensive process. Thus, an attenuated UPR saves cellular resources that are available for other cellular processes. 

We validated the expression system also with a range of different antibodies that featured different antigen binding domains and represented different antibody formats (Table 1). Furthermore, different signal sequences were used. Notably, the C2B8 antibody expressed from pEK5 carried the original DNA sequence used for expression in mammalian cells. Thus, the yeast cultivation set-up could also be utilized as an initial expression platform for antibodies that will later be expressed with mammalian cells. The cultivation system enables rapid screening of candidate antibodies and small-scale production of candidate molecules. The time from availability of an expression construct to the produced antibody at low µg/well quantities is around one week. Moreover, if multiple wells or a full plate is used, up to 100 to 150 µg can be produced. This concept of using yeast, in this case *P*. *pastoris*, for rapid prototyping of a VHH-Fc fusion was intriguingly demonstrated by Schepens et al. (2021).

Compared to the expression of the compact nanobodies, the titers for the chimeric nanobody-Fc fusion obtained in this study are still considerably lower (Frenken et al. 2000; Gorlani et al. 2012). However, the expression of nanobodies benefits from their smaller size, more hydrophilic framework, and higher solubility compared to antibodies carrying the Fc-domain, leading to improved expression (Bannas et al. 2017). Lower expression levels, along with reduced growth and altered cellular physiology, are well-documented phenomena for the expression of more complex antibody formats in *S*. *cerevisiae* (de Ruijter et al. 2017). Moreover, it must be kept in mind that production titers for different nanobodies in *S*. *cerevisiae* can be highly variable, a phenomena that could also apply to fusion constructs as expressed here (Gorlani et al. 2012). In yeast, to our knowledge, the expression of chimeric nanobody-Fc fusion was limited so far to the methylotrophic yeast *P*. *pastoris* (for example: Ji et al. 2013; Schepens et al. 2021; Zhang et al. 2021). However, the physiological differences between *S*. *cerevisiae* and *P*. *pastoris* make any comparison of the performance of these production organisms difficult.

Overall, the integrated cell engineering and medium optimization approach enabled the production of a chimeric nanobody-Fc fusion at microgram per milliliter scale using a small-scale cultivation set-up. When using a genetically modified strain, antibody titers of up to 2.55 µg/mL were obtained. In addition to a substantial improvement in antibody secretion, the produced antibodies were confirmed as functional by the lysozyme binding assay and the Fc-LC ELISA. These findings demonstrate that our approach increases antibody yield while preserving quality.

## Supplementary Information

Below is the link to the electronic supplementary material.

ESM 1 (DOCX 586 KB)

## Data Availability

All data pertaining to the findings of this study are available within this paper. Unprocessed raw data is provided upon request. All materials are shared with academic institutions upon request and execution of a material transfer agreement.
