# Supplementary material for: An integrated cell and medium engineering approach for production of a nanobody fusion in Saccharomyces cerevisiae
Source: Appl Microbiol Biotechnol. 2026 Jan 29;110:46. doi: 10.1007/s00253-025-13700-1 (PMC12858493; doi:10.1007/s00253-025-13700-1)
Supplement: Supplementary file 1 [file 253_2025_13700_MOESM1_ESM.docx]

Supporting information for:

**Applied Microbiology and Biotechnology**

**An integrated cell and medium engineering approach for production of a nanobody fusion in *Saccharomyces* *cerevisiae***

**Laura R. K. Niemelä, Lotta-Mari Kirjavainen, Hendrikje C. J. Kozlowski, Heidi Salminen and Alexander D. Frey**

Aalto University, Department of Bioproducts and Biosystems, Espoo, Finland

## Supplementary tables

**Supplemental Table S1.** Oligonucleotides used in this study

| Name | Target gene | DNA sequence 5´-3´ |
| --- | --- | --- |
| OAF55 |  | aaACTAGTATGAGGCAGGTTTGGTTCTCTTGGATTGTGGGATTGTTCCTATGTTTTTTCAACGTGTCTTCTGCTGATATCGTTTTGACTCAATCTCC |
| OAF56 |  | aaCTCGAGTTATTACTTACCTGGAGAC |
| EK003 | *YPS1* | ATGAAACTGAAAACTGTAAGATCTGCGGTCCTTTCGTCACTCTTTGCATCGTACGCTGCAGGTCGACAAC |
| EK004 | *YPS1* | TCAGATGAATGCAAAAAGAAGAGAAATTAATGTGAGGGGTAAAGATGGAACCACTAGTGGATCTGATATC |
| EK005 | *PRB1* | ATGAAGTTAGAAAATACTCTATTTACACTCGGTGCCCTAGGGAGCATCTCGTACGCTGCAGGTCGACAAC |
| EK006 | *PRB1* | TTAAATAATATTCAATTTATCAAGAATATCTCTCACTTGATCAAAGATTACCACTAGTGGATCTGATATC |
| OAF104 | *PEP1* | ATGATATTACTTCATTTTGTCTATTCTCTTTGGGCCTTACTTCTCATTCCGTACGCTGCAGGTCGACAAC |
| OAF105 | *PEP1* | CTACTGGTTTTCGTTAGATGGCGCTGTAGAATCAGGCCTGTCGATGTTTTGCATAGGCCACTAGTGGATCTG |
| OAF106 | *MON2* | ATGGCCATGAACACTGGAGGGTTTGACTCCATGCAAAGGCAACTTGAAGCGTACGCTGCAGGTCGACAAC |
| OAF107 | *MON2* | CTAGTCTAGTTTCGTAAATCCAAGTGATAATTCCAAAACTTTATCTTGGAGCATAGGCCACTAGTGGATCTG |
| OAF108 | *ALG3* | ATGGAAGGTGAACAGTCTCCGCAAGGTGAAAAGTCTCTGCAAAGGAAGCAGTACGCTGCAGGTCGACAAC |
| OAF109 | *ALG3* | TCAGTTGAGCTTTTTTTCCATAGAGCTGGTGGTACGAAGATGGCTTTTGGGCATAGGCCACTAGTGGATCTG |
| OJR38 | *PEP4* | ATGTTCAGCTTGAAAGCATTATTGCCATTGGCCTTGTTGTTGGTCAGCGCGAGGCCCAGAATACCCTCC |
| OJR39 | *PEP4* | TCAAATTGCTTTGGCCAAACCAACCGCATTGTTGCCCAAATCGTAAATAGCACTGGATGGCGGCGTTAG |
| OJR72 | *VPS30* | ATGAAGTGCCAAACATGTCACTTACCCCTGCAACTAGACCCATCTTTAGACAGCTGAAGCTTCGTACGC |
| OJR73 | *VPS30* | TTAGTTTCCGCTGATGGTCTTATCATTGTAATTCACTGTAGGGCTTAAGGGCATAGGCCACTAGTGGATCTG |
| OMP42 | *PEP4* | GTGACCTAGTATTTAATCCAAATAAAATTCAAACAAAAACCAAAACTAACGTACGCTGCAGGTCGACAAC |
| OMP43 | *PEP4* | CTCTCTAGATGGCAGAAAAGGATAGGGCGGAGAAGTAAGAAAAGTTTAGCGCATAGGCCACTAGTGGATCTG |
| EK158 | *PRB1*con | CTCAGTAATGCCACTGCAG |
| OJR29 | *PEP1*con | CCTCTCCCAGTAACGACTTC |
| OJR30 | *CYM1*con | GCCATGAACAACCTTCT |
| OJR31 | *MON2*con | CACGGTATTACGGGTTTACG |
| OJR32 | *ALG3*con | GACCAGCTTGGTTATGTG |
| OJR40 | *PEP4*con | AATGCGAATACGGGGAAC |
| OJR78 | *VPS30*con | TCTGGTTCGTAGGCTTCTTC |
| OJR95 | KanMxfor | TTGATGACGAGCGTAATGGC |
| OJR96 | KanMxrev | AAGATCCTGGTATCGGTCTG |
| OJR61 | incasrev | GGATGTATGGGCTAAATG |

**Supplemental Table S2.** Screening of chemical chaperones and osmolytes

| **Compounds** | **Concentration range tested** | **Effects^a)^ on growth** | **Effects on titer** | **Reference** |
| --- | --- | --- | --- | --- |
| Arginine | 1 mM - 25 mM | +/- | ++ | Kang et al. 2000; Sharma et al. 2013 |
| Tween-20 | 0.001% - 0.005% | +/- | + | Baumann et al. 2011 |
| Sodium 4-phenylbutyrate (4-PBA) | 0.1 mM - 5.0 mM | +/- | ++ | Johari et al. 2015 |
| Dithiotreitol (DTT) | 0.5 mM - 2.0 mM | - | - | Frand and Kaiser 1998 |
| Glycerol | 1.0% - 8% | -- | -- | Figler et al. 2000; Street et al. 2006; Johari et al. 2015 |
| CaCl_2_ | 0.0025 mM - 0. 25 mM | - | + | Dulary et al. 2018 |
| MnCl_2_ | 0.01 mM - 1.0 mM | - | + | Dulary et al. 2018 |
| CuSO_4_ | 0.001 - 0.25 mM | - | + | Frand and Kaiser 1998 |
| Glucose-6-phosphate | 0.1 - 10 mM | +/- | + | This study |
| Adenosine triphosphate (ATP) | 0.02 - 5.0 mM | - | + | This study |
| Dimethylsulfoxide (DMSO) | 0.1% - 1.0% | - | - | Sadowska-Bartosz et al. 2013; Johari et al. 2015 |
| Triton X-100 | 0.01% - 0.1% | -- | -- | Baumann et al. 2011 |
| Alanine | 5 mM - 25 mM | -- | +/- | Dandage et al. 2015 |
| Proline | 5 mM - 25 mM | +/- | +/- | Street et al. 2006; Dandage et al. 2015 |
| Glutamic acid | 0.5 mM - 5 mM | +/- | + | Wu et al. 2013 |
| Glycine | 5 mM - 150 mM | - | +/- | Dandage et al. 2015 |
| Betaine | 10 - 150 mM | +/- | +/- | Street et al. 2006; Johari et al. 2015 |
| Taurine | 10 mM - 150 mM | - | - | Abe et al. 2015 |
| Diamide (tetramethylazodicarboxamide, TMAD) | 0.2 mM - 50 mM | -- | -- | Frand and Kaiser 1998 |
| Trimethylamine *N*-oxide (TMAO) | 2 mM - 100 mM | -- | - | Street et al. 2006; Johari et al. 2015 |
| Dimethylethylammoniumpropane sulfonate (NDSB 195) | 1 mM - 100 mM | +/- | +/- | Mizukoshi et al. 2015 |
| 1-(3-sulfopropyl)pyridinium betaine (NDSB 201) | 2 - 10 mM | +/- | +/- | Willis et al. 2005 |

**^a)^** Effect: increase/decrease < 10%: + or -; increase/decrease 10 - 30%: ++ or --; increase/decrease > 30%: +++ or ---; no change: +/-

## Supplemental figures

**Supplemental Fig S1** Original, non-cropped images of the immunoblot analysis of chimeric nanobody-Fc fusion proteins expressed in control and deletion yeast strains. Non-reduced (- DTT), reduced (+ DTT), and reduced deglycosylated (+ DTT, + EndoH) samples are shown. Lane 6 contains the trace of an additional strain not included in the study. The loading control is the heavy chain of a reduced human IgG_1_ antibody. Left side, overlay of immunoblot and true colour image of membrane to who; right side: immunoblot.

**References**

Abe Y, Ohkuri T, Yoshitomi S, Murakami S, Ueda T (2015) Role of the osmolyte taurine on the folding of a model protein, hen egg white lysozyme, under a crowding condition. Amino Acids 47:909–915. https://doi.org/10.1007/s00726-015-1918-0

Baumann K, Adelantado N, Lang C, Mattanovich D, Ferrer P (2011) Protein trafficking, ergosterol biosynthesis and membrane physics impact recombinant protein secretion in *Pichia pastoris*. Microb Cell Fact 10:93. https://doi.org/10.1186/1475-2859-10-93

Dandage R, Bandyopadhyay A, Jayaraj GG, Saxena K, Dalal V, Das A, Chakraborty K (2015) Classification of chemical chaperones based on their effect on protein folding landscapes. ACS Chem Biol 10:813–820. https://doi.org/10.1021/cb500798y

Dulary E, Yu SY, Houdou M, de Bettignies G, Decool V, Potelle S, Duvet S, Krzewinski-Recchi MA, Garat A, Matthijs G, Guerardel Y, Foulquier F (2018) Investigating the function of Gdt1p in yeast Golgi glycosylation. Biochim Biophys Acta Gen Subj 1862:394–402. https://doi.org/10.1016/j.bbagen.2017.11.006

Figler RA, Omote H, Nakamoto RK, Al-Shawi MK (2000) Use of chemical chaperones in the yeast *Saccharomyces cerevisiae* to enhance heterologous membrane protein expression: High-yield expression and purification of human P-glycoprotein. Arch Biochem Biophys 376:34–46. https://doi.org/10.1006/abbi.2000.1712

Frand AR, Kaiser CA (1998) The *ERO1* gene of yeast is required for oxidation of protein dithiols in the endoplasmic reticulum. Mol Cell 1:161–170

Johari YB, Estes SD, Alves CS, Sinacore MS, James DC (2015) Integrated cell and process engineering for improved transient production of a “difficult-to-express” fusion protein by CHO cells. Biotechnol Bioeng 112:2527–2542. https://doi.org/10.1002/bit.25687

Kang HA, Choi E-S, Hong W-K, Kim J-Y, Ko S-M, Sohn J-H, Rhee SK (2000) Proteolytic stability of recombinant human serum albumin secreted in the yeast *Saccharomyces cerevisiae*. Appl Microbiol Biotechnol 53:575–582

Mizukoshi Y, Takeuchi K, Arutaki M, Takizawa T, Hanzawa H, Takahashi H, Shimada I (2015) Suppression of problematic compound oligomerization by cosolubilization of nondetergent sulfobetaines. Chem Med Chem 10:736–741. https://doi.org/10.1002/cmdc.201500057

Sadowska-Bartosz I, Paczka A, Moloń M, Bartosz G (2013) Dimethyl sulfoxide induces oxidative stress in the yeast *Saccharomyces cerevisiae*. FEMS Yeast Res 13:820–830. https://doi.org/10.1111/1567-1364.12091

Sharma S, Sarkar S, Paul SS, Roy S, Chattopadhyay K (2013) A small molecule chemical chaperone optimizes its unfolded state contraction and denaturant like properties. Sci Rep 3:3525 https://doi.org/10.1038/srep03525

Street TO, Wayne Bolen D, Rose GD, Jenkins TC (2006) A molecular mechanism for osmolyte-induced protein stability. Proc Natl Acad Sci 103:13997–14002

Willis MS, Hogan JK, Prabhakar P, Liu X, Tsai K, Wei Y, Fox T (2005) Investigation of protein refolding using a fractional factorial screen: A study of reagent effects and interactions. Protein Sci 14:1818–1826. https://doi.org/10.1110/ps.051433205

Wu Z, Song L, Liu SQ, Huang D (2013) Independent and additive effects of glutamic acid and methionine on yeast longevity. PLoS One 8. https://doi.org/10.1371/journal.pone.0079319
